# Effect of within-endobag method during laparoscopic ovarian cystectomy of dermoid cyst: A retrospective study

**DOI:** 10.1097/MD.0000000000033043

**Published:** 2023-02-17

**Authors:** Jisun Lee, Hee Jeong Kim, Yujin Heo, Hyun Jung Lee

**Affiliations:** a Department of Obstetrics and Gynecology, School of Medicine, Kyungpook National University, Daegu, Republic of Korea; b Department of Obstetrics and Gynecology, Kyungpook National University Hospital, Daegu, Republic of Korea.

**Keywords:** cystectomy, dermoid cyst, endobag, laparoscopy

## Abstract

This study aimed to evaluate the effect of within-endobag cystectomy during and after laparoscopic ovarian cystectomy in patients with dermoid cysts. We retrospectively analyzed 84 patients with ovarian dermoid cysts who underwent laparoscopic ovarian cystectomy. In 30 patients, the affected ovary was placed in an endobag before cystectomy and cystectomy was performed within an endobag (within-endobag group), while the remaining 54 patients underwent standard cystectomy without this step (without-endobag group). After cystectomy, the cyst wall was placed in an endobag and was removed from the abdomen. Compared with the without-endobag group, the within-endobag group had a significantly lower rate of cyst content spillage (23.3% vs 72.2%, *P* < .001) and significantly shorter operation times when the cysts ruptured (23.4 ± 8.6 minutes vs 51.2 ± 28.6 minutes, *P* < .001). Whereas there was no significant difference in operation time in the absence of cyst rupture between 2 groups (21.2 ± 8.8 minutes vs 31.1 ± 17.4 minutes, *P* = .111). In patients with cyst rupture, according to the cyst size increase, the operation time was significantly prolonged without-endobag, whereas no significant prolongation was observed in within-endobag cystectomy. Except for operation time, there were no significant differences in cyst length, pain on the first day after surgery, hemoglobin loss, hospital stay, and inflammatory markers (C-reactive protein and white blood cell counts) in both ruptured and unruptured cases between the 2 groups. There were no postoperative complications in the within-endobag group, but 2 cases of perioperative complications occurred in the without-endobag group. No chemical peritonitis due to spillage of the cyst contents was observed in either group. Laparoscopic ovarian cystectomy performed within-endobag can reduce both the spillage rate of cyst contents and operation time regardless of cyst size in patients with ruptured cysts. Therefore, this technique is a good surgical option for the laparoscopic ovarian cystectomy of large dermoid ovarian cysts.

## 1. Introduction

Ovarian dermoid cysts are one of the most common benign ovarian neoplasms in women of reproductive age.^[1]^ Laparoscopic ovarian cystectomy is preferred over traditional laparotomy because of less postoperative pain, less adhesion, lower infection rates, and shorter duration of hospital stay.^[2,3]^ However, despite the advantages of laparoscopic surgery, concerns remain about inadvertent cyst rupture and the resulting spillage of the cyst contents. The release of sebaceous fluid with hair, fat, bone, cartilage, and blood may be related to postoperative chemical peritonitis, pelvic adhesions, infertility, and cyst recurrence.^[3,4]^

The use of an endobag to remove the ovarian cyst wall from the abdominal cavity after laparoscopic cystectomy seems to be effective in decreasing the chance of spillage of cyst contents during cyst wall extraction.^[5]^ Although this technique is now considered an essential method in ovarian cyst surgery, it is not a preventative method to reduce spillage of cyst contents that may occur during cystectomy itself. Therefore, to minimize spillage during cystectomy, a surgical technique within the bag has been introduced.^[6–8]^ However, most studies have used an endoscopic retrieval bag to minimize the risk of intraoperative spillage of the ovarian cyst content during removal of the cyst wall from the abdominal cavity.^[3,8–10]^ In this study, we evaluated the effects of within-endobag cystectomy that performing the entire cystectomy procedure within an endobag by comparing the surgical and postoperative clinical data with those of a group of patients with ovarian dermoid cysts who did not use an endobag during cystectomy.

## 2. Materials and methods

### 2.1. Study design and participants

We retrospectively analyzed 84 patients with ovarian dermoid cysts who underwent laparoscopic ovarian cystectomy between July 2017 and October 2021 at the Department of Obstetrics and Gynecology of Kyungpook National University Hospital, Daegu, Korea. The diagnosis of ovarian dermoid cysts was established preoperatively by pelvic examination, ultrasonography, or computed tomography and was confirmed histologically. Patients were excluded if they had a history of intraabdominal surgery, evidence of malignancy, or a combination of other ovarian tumors. The institutional review board of Kyungpook National University Hospital approved the study and waived the requirement of obtaining informed consent owing to the study retrospective design (approval number: 2022-05-018). All procedures were performed in accordance with the latest version of the declaration of Helsinki.

### 2.2. Procedure

**Figure 1.**
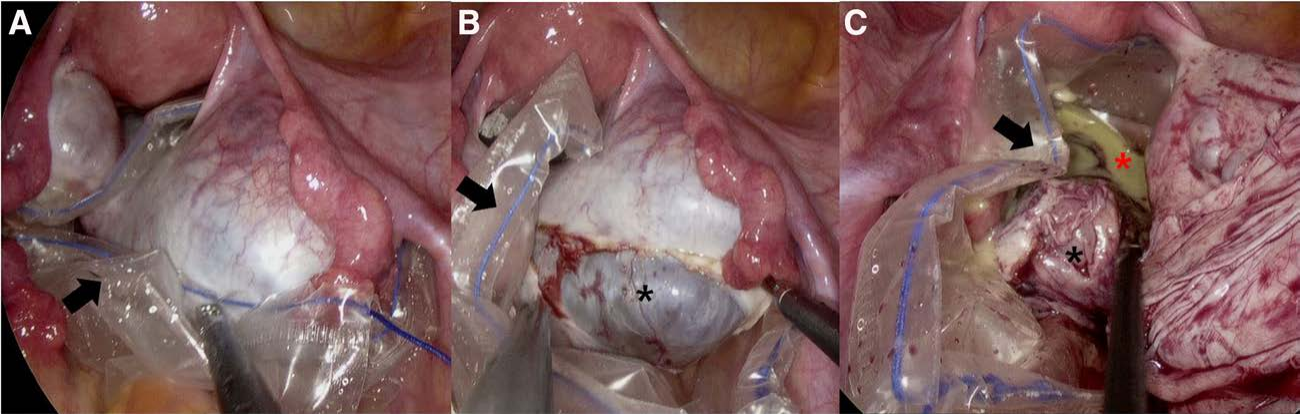
In within-endobag group, before starting cystectomy, the memory wire of endobag was removed and the affected ovary was placed within an endobag (arrow) (a). After the identification of the cleavage plane between the cyst capsule and the adjacent normal ovarian tissue, the cyst wall (black asterisk) was stripped (b). In cases with cyst rupture, the cyst contents (red asterisk) were contained in the endobag without spillage to abdominal cavity (c).

**Figure 2.**
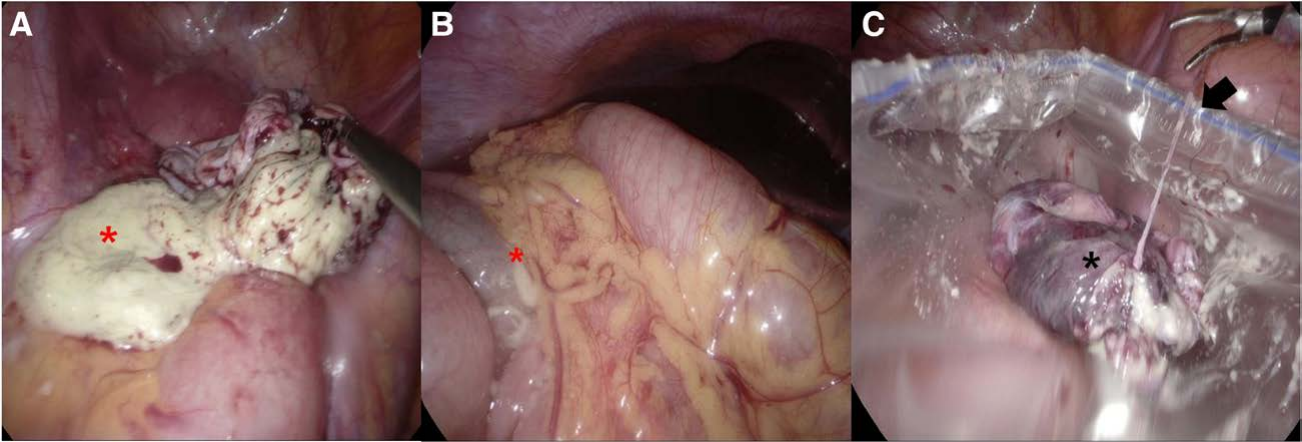
In without-endobag group, cyst contents (red asterisk) could flood the pelvic (a) and upper abdominal cavity (b). The cyst wall (black asterisk) was removed from abdominal cavity using the endobag (arrow) (c).

All the patients underwent laparoscopic ovarian cystectomy. A multichannel, single-port trocar was inserted through the umbilicus. One or 2 ancillary trocars were inserted in the lower quadrant of the abdomen depending on the cyst size. Initially, the pelvis and abdomen were examined to assess adhesion or the risk of malignancy. Patients who underwent cystectomy within an endobag comprised the within-endobag group (N = 30). In this group, before starting cystectomy, an endobag was inserted through the umbilical port and the memory wire of the endobag was removed. The affected ovary was placed and fully wrapped in an endobag (Fig. 1a), and cystectomy was performed within the endobag. An incision was made on the antimesenteric border of the ovary using a monopolar hook. After identifying the cleavage plane between the cyst capsule and the adjacent normal ovarian tissue, the cyst wall was stripped using 2 atraumatic grasping forceps (Fig. 1b). In cases with cyst rupture, the cyst contents were contained in the endobag without spillage into the abdominal cavity (Fig. 1c). After enucleation, the cyst wall was removed from the abdominal cavity by using the same endobag. Patients who underwent cystectomy without an endobag comprised the without-endobag group (N = 54). Without-endobag cystectomy, cyst contents could flood the pelvis (Fig. 2a) and the upper abdominal cavity (Fig. 2b). After cystectomy, the cyst wall was placed in an endobag (Fig. 2c) and removed from the abdomen by the same endobag. If rupture of the cyst capsule occurred, the sebaceous material was promptly aspirated, taking care to minimize spillage and peritoneal contamination. Multiple suctions and washings were performed using a suction irrigation cannula until the rinsing fluid became clear. In all patients, the abdominal cavity was washed with saline solution at the end of the surgery. If necessary, hemostasis was achieved using bipolar forceps.

### 2.3. Definitions

Operative time was defined as the time from immediately after inspection of the abdomen through removal of the ovarian cyst wall from the abdominal cavity to the end of suction and irrigation. The cyst length was defined as the maximum diameter of the cyst on ultrasonographic examination. Hemoglobin loss was defined as the difference in hemoglobin levels before surgery and on the first day after surgery. C-reactive protein (CRP) and white blood cell counts (WBCs) were examined as acute inflammatory markers. Differences in CRP levels and WBCs were defined as the differences in CRP levels and WBCs before surgery and on the first day after surgery. The pain score on the first day after surgery was measured using a visual analog scale.

### 2.4. Statistical analysis

Statistical analyses, including logistic regression analysis, were performed using a commercial software package (SPSS for Windows, v13.0; SPSS, Chicago, IL). Statistical significance was set at *P* < .05. Student *t* test was used to examine the differences in numerical variables. Categorical variables were described as percentages and compared using the Pearson chi-square or Fisher exact test, as appropriate.

## 3. Results

The clinical characteristics of the study participants are summarized in Table 1. The patients demographic characteristics and perioperative outcomes were comparable between the groups. No significant differences were observed in acute inflammatory response markers (CRP level and WBCs) before and after surgery. Cysts ruptured during cystectomy in 10 cases (33%) and 38 cases (70.4%) in the within-endobag and without-endobag groups, respectively, although the difference was not statistically significant (*P* = .454). However, compared with the without-endobag group, the within-endobag group had significantly less spillage of cyst contents (23.3% vs 72.2%; *P* < .001) and significantly shorter operation times for cystectomy (22.7 ± 8.6 minutes vs 45.4 ± 27.3 minutes; *P* < .001). Neither group experienced intraoperative complications or chemical peritonitis, whereas only the within-endobag group experienced no postoperative complications. The without-endobag group had 1 case of paralytic ileus and 1 case of rehospitalization due to abdominal pain (Table 1).

**Table 1:** Clinical characteristics and perioperative data in patients with ovarian dermoid cyst who underwent laparoscopic ovarian dermoid cystectomy.

	Within-endobag	Without-endobag	*P* value
	(n = 30)	(n = 54)	
Age (yr)	29.5 ± 8.3	28.2 ± 7.4	.470
BMI (kg/m^2^)	20.8 ± 2.5	22.6 ± 4.3	.016
Operative time (min)	22.7 ± 8.6	45.4 ± 27.3	<.001
Parity	0.3 ± 0.7	0.2 ± 0.5	.533
Hospital stays (d)	3.0 ± 0.7	3.2 ± 0.8	.262
Cyst length (mm)	73.9 ± 44.0	60.8 ± 25.5	.141
Trocar number (n)	1.5 ± 0.5	2.1 ± 0.9	.001
Hemoglobin loss (g/dL)	1.6 ± 0.7	1.5 ± 0.7	.449
DWBCs (10^3^/µL)	2.6 ± 1.5	2.6 ± 1.3	.469
DCRP (mg/dL)	1.2 ± 1.0	1.7 ± 1.4	.141
Pain on first d after surgery, VAS score	2.8 ± 1.0	3.0 ± 0.7	.273
Cyst rupture rate (%)	66.7	70.4	.454
Spillage rate (%)	20.0	70.4	<.001
Adhesion rate (%)	23.3	16.7	.320
Drain use (%)	60.0	59.3	.567
Bilaterality (%)	3.3	11.1	.210
Postoperative Complications (n)	0	2	

DCRP = difference of C-reactive protein, DWBCs = difference of white blood cell counts, VAS = visual analogue scale.

**Figure 3.**
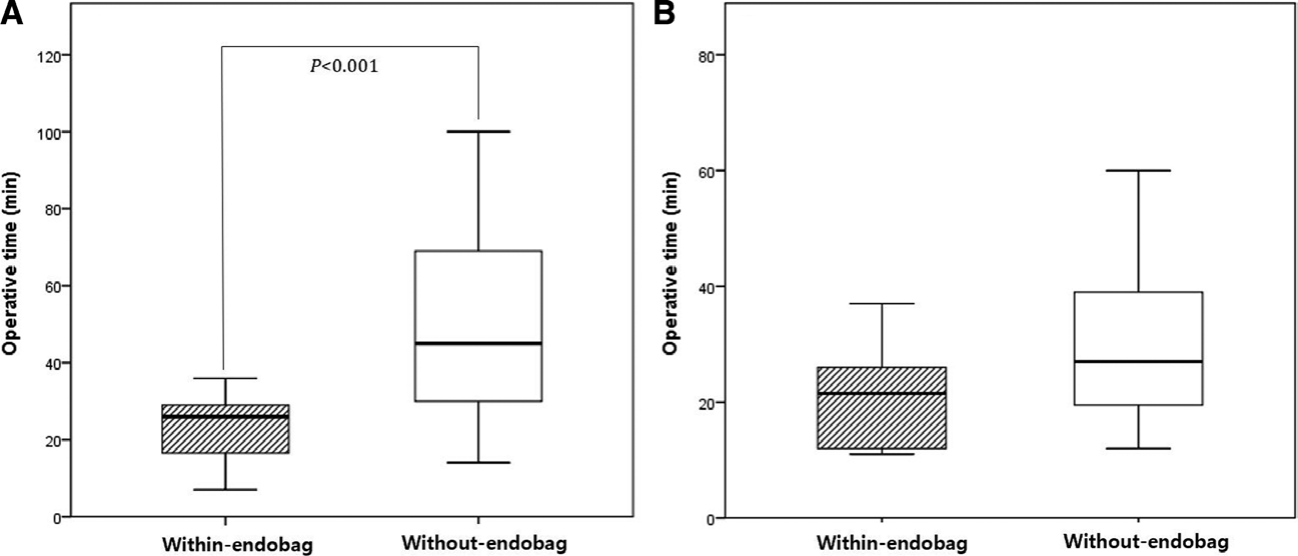
Operative time was compared between within-endobag and without-endobag group each in cases with cyst ruptured (a) and unruptured (b). In cases with cyst rupture (a), within-endobag group (striped bar) showed significantly shorter operative time (*P* < .001). Whereas there was no significant difference in operation time in the absence of cyst rupture (b).

**Figure 4.**
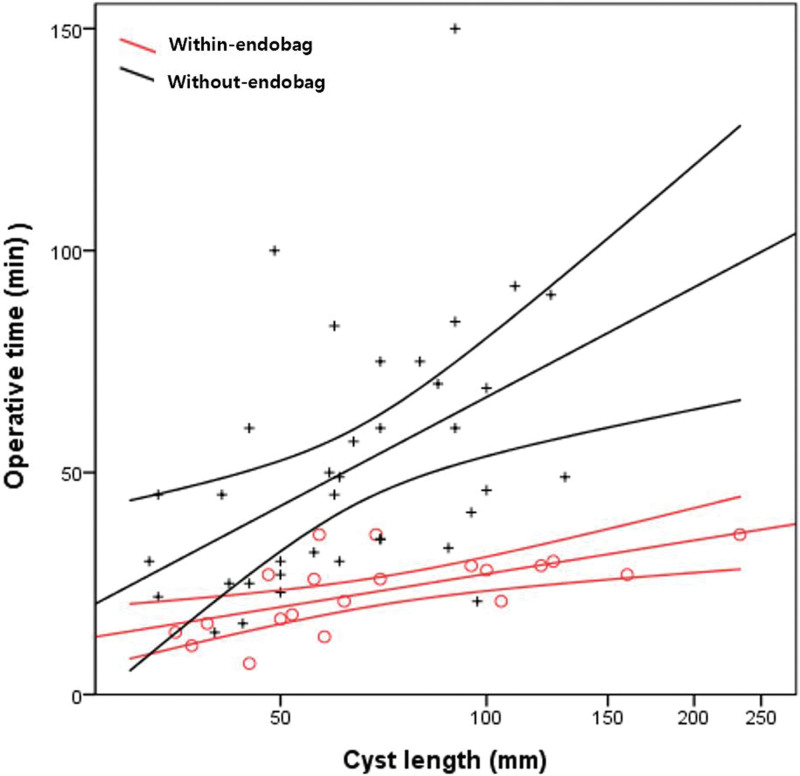
Linear regression analysis between cyst size and operation time. There was no significant prolongation of the operation time even if the size increased in within-endobag group (red, R^2^ = 0.422), whereas in without-endobag group (black, R^2^ = 0.221), the operation time was significantly prolonged as the size increased (*P* < .001).

When cyst rupture occurred during cystectomy (n = 58), the within-endobag group had significantly shorter operation times than the without-endobag group (23.4 ± 8.6 minutes vs 51.2 ± 28.6 minutes; *P* < .001) (Fig. 3a). In the absence of cyst rupture, we observed no significant difference in operation times between the 2 groups (21.2 ± 8.8 minutes vs 31.1 ± 17.4 minutes; *P* = .111) (Fig. 3b).Except for operation time, there were no significant differences in cyst length, pain on the first day after surgery, hemoglobin loss, hospital stay, and inflammatory markers (CRP and WBCs) between the 2 groups, irrespective of cyst rupture (Table 2). The operation time for cystectomy was correlated with cyst size in both groups when cyst ruptured. However, prolongation of the operation time was not observed even when the size increased within-endobag group (R^2^ = 0.422), whereas in without-endobag group, the operation time was significantly prolonged as the size increased (R^2^ = 0.221) (*P* < .001) (Fig. 4).

**Table 2:** Comparison of perioperative data between within-endobag and without-endobag group each in cases with cyst ruptured and unruptured.

	R`uptured			Unruptured		
	Within-endobag n=20	Without-endobag n=38	*p*-value	Within-endobag n=10	Without-endobag n=16	*p*-value
Operative time (min)	23.4 ± 8.6	51.2 ± 28.6	<.001	21.2 ± 8.8	31.1 ± 17.4	.111
Cyst length (mm)	80.8 ± 49.4	67.9 ± 25.7	.285	60.1 ± 28.0	43.8 ± 15.21	.066
Trocar number (n)	1.6 ± 0.5	2.3 ± 0.9	.002	1.3 ± 0.5	1.8 ± 0.8	.114
Pain on the first d after surgery, VAS score	2.8 ± 1.0	3.1 ± 0.7	.226	2.7 ± 0.8	2.8 ± 0.7	.868
Hemoglobin loss (g/dL)	1.6 ± 0.7	1.5 ± 0.7	.473	1.5 ± 0.6	1.5 ± 0.4	.780
DWBCs (10^3^/µL)	2.8 ± 1.5	2.3 ± 1.9	.308	2.0 ± 2.2	2.1 ± 1.8	.918
DCRP (mg/dL)	1.2 ± 1.2	1.7 ± 0.8	.698	1.0 ± 0.6	1.8 ± 1.7	.289
Hospital stays (d)	3.1 ± 0.8	3.2 ± 0.7	.527	2.8 ± 0.4	3.1 ± 1.0	.318

DCRP = difference of C-reactive protein, DWBCs = difference of white blood cell counts, VAS = visual analogue scale.

## 4. Discussion

We evaluated the effects of within-endobag cystectomy which is performing the entire cystectomy procedure within an endobag during the laparoscopic ovarian dermoid cystectomy. In this study, it was confirmed that within-endobag cystectomy can reduce both the spillage rate of cyst contents and operation time regardless of cyst size in patients with ruptured cysts.

Laparoscopic surgery has been widely used for the treatment of benign ovarian tumors because of the many advantages of a minimally invasive approach; however, it can also cause complications such as chemical peritonitis or spread of infection related to the spillage of cyst contents during surgery.^[9–12]^ The incidence of chemical peritonitis after rupture or leakage of cystic teratomas is < 0.5%.^[13]^ Surgical techniques such as the use of endobag or purse-string suture have been introduced to prevent complications associated with leakage of cyst contents.^[6–8,14]^ In cases of cyst rupture, jet-wash irrigation with large amounts of fluid and removal of tiny particles of cyst contents is considered the gold standard procedure to avoid complications, but it has the disadvantage of requiring a long time.^[15,16]^ Despite the rare incidence of short-and long-term outcomes associated with intraoperative spillage, surgeons should make efforts to prevent intraperitoneal spillages. Therefore, there is a need for a method to avoid spillage and reduce operation time had been raised. In the within-endobag procedure described in this study, the entire affected ovary was placed in the endobag before performing cystectomy. An endobag was inserted through the umbilicus and the memory wire of the endobag was removed. This step allows the endobag to sufficiently envelop the ovary and reduce the collision between the instrument and endobag inserter during cystectomy. To minimize leakage, an endobag of sufficient size was used to contain the ovaries completely. The Trendelenburg position used in laparoscopy increases the likelihood that the cyst contents will enter the upper abdominal cavity, so the entrance of the endobag was raised sufficiently towards the inlet of the pelvis to allow the contents to flow towards the cul-de-sac. The bottom of the endobag was directed to the cul-de-sac and then spread out sufficiently to allow the cyst contents to descend into the cul-de-sac during cyst rupture.

In this study, patients who underwent within-endobag cystectomy had significantly reduced spillage rates and operation times compared with those who underwent without-endobag cystectomy. Although patients in the without-endobag group experienced no increase in complication rates or acute inflammatory marker levels when cyst contents entered the abdominal cavity, they required more time for aspiration and irrigation to remove the cyst contents. Moreover, while the operation time did not differ as cyst size increased in the within-endobag group, we observed significantly increased operation times as cyst size increased in the without-endobag group. This probably reflects the increased amount of cyst contents flowing into the abdominal cavity when not using an endobag, with removal of the cyst contents often requiring more time than the cystectomy itself. When cyst rupture occurred during the within-endobag procedure, less time was required to remove the cyst contents from the abdominal cavity, allowing for significantly shorter operation times.

Despite observing no significant differences in rupture rates by endobag use, we found that the spillage rate was significantly lower in the within-endobag group than in the without-endobag group. When performing cystectomy without an endobag, spillage into the abdominal cavity occurs as an inevitable consequence of cyst rupture, whereas within-endobag cystectomy can prevent or reduce spillage. Although our sample size was small, there were no reported cases of chemical peritonitis, and it was confirmed that our method reduced the spillage rate and operation time. Our study has some limitations. First, this was not a prospective randomized clinical trial, the sample size was relatively small. Postoperative complications were found in only 2 cases in which the endobag was not used, so no statistical difference could be confirmed. Therefore, additional studies and randomized clinical trials are recommended to confirm the clinical outcomes of this technique. Second, the surgeries were not performed by the same surgeon. The surgeon skills were not the same, which could affect the operation time. Surgeons with less experienced in laparoscopic surgery may have difficulty performing this procedure at first, but they will get used to it after several times and this procedure is easier and less time consuming than removing spilled cyst contents.

In conclusion, this study showed that within-endobag cystectomy can reduce both the spillage of cyst contents and the overall operation time during cystectomy, irrespective of cyst size, in patients with ruptured dermoid cysts. As the size of the cyst increased, the operation time of within-endobag group was within a certain range; therefore, it is considered a good surgical option for patients with large dermoid ovarian cysts.

## Author contributions

**Conceptualization:** Jisun Lee, Hyun Jung Lee.

**Data curation:** Hee Jeong Kim, Yujin Heo, Hyun Jung Lee.

**Formal analysis:** Hyun Jung Lee.

**Investigation:** Hee Jeong Kim, Hyun Jung Lee.

**Methodology:** Jisun Lee, Yujin Heo, Hyun Jung Lee.

**Project administration:** Hyun Jung Lee.

**Software:** Hee Jeong Kim.

**Supervision:** Hyun Jung Lee.

**Visualization:** Jisun Lee.

**Writing – original draft:** Jisun Lee, Hyun Jung Lee.

**Writing – review & editing:** Hee Jeong Kim, Yujin Heo, Hyun Jung Lee.
